# Basil functional and growth responses when cultivated via different aquaponic and hydroponics systems

**DOI:** 10.7717/peerj.15664

**Published:** 2023-07-18

**Authors:** Anastasia Mourantian, Maria Aslanidou, Eleni Mente, Nikolaos Katsoulas, Efi Levizou

**Affiliations:** 1 Department of Agriculture Crop Production and Rural Environment, University of Thessaly, N. Ionia, Volos, Greece; 2 Department of Veterinary Medicine, Aristotle University of Thessaloniki, Thessaloniki, Greece

**Keywords:** Photosynthesis, Leaf nutrients, Soilless cultivation, *Ocinum basilicum*, Red tilapia, PRI index, Plant physiology, CO_2_ assimilation, Chlorophyll content

## Abstract

**Background:**

Aquaponics is an innovative farming system that combines hydroponics and aquaculture, resulting in the production of both crops and fish. Decoupled aquaponics is a new approach introduced in aquaponics research for the elimination of certain system bottlenecks, specifically targeting the optimization of crops and fish production conditions. The aquaponics-related literature predominantly examines the system’s effects on crop productivity, largely overlooking the plant functional responses which underlie growth and yield performance. The aim of the study was the integrated evaluation of basil performance cultivated under coupled and decoupled aquaponic systems compared with a hydroponic one, in terms of growth and functional parameters in a pilot-scale aquaponics greenhouse.

**Methods:**

We focused on the efficiency of the photosynthetic process and the state of the photosynthetic machinery, assessed by instantaneous gas exchange measurements as well as photosynthetic light response curves, and *in vivo* chlorophyll *a* fluorescence. Light use efficiency was estimated through leaf reflectance determination. Photosynthetic pigments content and leaf nutritional state assessments completed the picture of basil functional responses to the three different treatments/systems. The plant’s functional parameters were assessed at 15-day intervals. The experiment lasted for two months and included an intermediate and a final harvest during which several basil growth parameters were determined.

**Results:**

Coupled aquaponics resulted in reduced growth, which was mainly ascribed to sub-sufficient leaf nutrient levels, a fact that triggered a series of negative feedbacks on all aspects of their photosynthetic performance. These plants experienced a down-regulation of PSII activity as reflected in the significant decreases of quantum yield and efficiency of electron transport, along with decreased photosynthetic pigments content. On the contrary, decoupled aquaponics favored both growth and photochemistry leading to higher light use efficiency compared with coupled system and hydroponics, yet without significant differences from the latter. Photosynthetic light curves indicated constantly higher photosynthetic capacity of the decoupled aquaponics-treated basil, while also enhanced pigment concentrations were evident. Basil functional responses to the three tested production systems provided insights on the underlying mechanisms of plant performance highlighting key-points for systems optimization. We propose decoupled aquaponics as an effective system that may replace hydroponics supporting high crops productivity. We suggest that future works should focus on the mechanisms involved in crop and fish species function, the elucidation of which would greatly contribute to the optimization of the aquaponics productivity.

## Introduction

The necessity for sustainable food production has been widely recognized as a challenge and a target for the current era, in order to tackle the problems arising from the natural resources over-exploitation and simultaneously feed an expanding world population. Concerning agriculture, the minimization of water consumption and chemical fertilizers input are crucial components of sustainable crops production (Krastanova et al., 2022). Hydroponics is the most intensive soilless production system in horticultural industry, which can succeed significant yields using lower amount of water comparatively to soil (Gruda, 2009). New, high tech and sophisticated greenhouses have accomplished the target of considerable water and nutrient re-use, thus reducing their ecological footprint (Elvanidi et al., 2020). However, hydroponics is a resource-demanding system, especially in terms of high inputs of chemical fertilizers, which compromise their sustainability. Aquaponics is a production system that addresses all the above-mentioned issues by combining the cultivation of plants in a soilless setup with the rearing of aquatic organisms in recirculating aquaculture systems (RAS) (Goddek et al., 2019). The recirculating water between the two sub-systems transfers the nitrogen-rich products of fish metabolism, uneaten feed and dissolved organic debris to the plants that utilize them as nutrients. Therefore it is considered a highly sustainable system due to the use of bio-nutrients instead of chemical supplementations, and the minimization of the water demands, which may reach a 10% of the amount needed in conventional soil cultivation (Bernstein, 2011). Additionally, aquaponics is virtually a circular economy concept as fish waste is transformed into resource. Krastanova et al. (2022) in a thorough analysis of the biological and technological parameters of aquaponics systems characterize them as specific mini-ecosystems, analogous to natural processes due to the symbiotic and synergistic environment of their function.

In a typical coupled aquaponic system, the aquaculture and the hydroponic units are arranged in a single loop thus the water flows continuously from the fish tanks to the plant unit and back, providing a constant supply of nutrients to plants (Yep & Zheng, 2019). This symbiotic environment is based on a compromise between different requirements of the two biological systems, fish and plant, concerning the environmental conditions and water chemistry parameters. The most crucial factor is the different pH optima, as fish require high pH values (7–9), in contrast to the 5.5−6.5 range of plants (Suhl et al., 2016). pH determines the availability of nutrients for absorption by plants, through its influence on the solubility and chemical form of the ions present in the water, and additionally on the events in the rhizosphere during absorption (Hinsinger et al., 2003). If the pH of a coupled aquaponic system is kept high satisfying the requirements of fish, then a series of essential nutrients such as iron and phosphorus, among others, would form insoluble salts and precipitate (Diaz, Reddy & Moore, 1994; Roosta, 2011). The resulting unavailability is mirrored in plant nutrient status, with nutrient deficiencies limiting growth and crop productivity. A compromise between the different requirements is always followed, resulting to pH adjusted around 7 (Saha, Monroe & Day, 2016; Vandam et al., 2017). Although compromises like this ensure the functionality of the whole system, their inevitable deviation from plant optima prevents high productivity in coupled aquaponic systems (Zou et al., 2016; Monsees, Kloas & Wuertz, 2017). Another source of nutrient limitation in coupled aquaponics is the fact that the only external input is fish feed, which poses two types of problems. First, the composition of feed meets the fish needs but may contain low concentrations of essential nutrients for plants due to low levels required for fish growth, as in the case of potassium. Second, the nutrients contained in the feed are primarily absorbed by fish and only part of them is subsequently released in the water to be available for plants. All the above-mentioned issues collectively result in sub-optimal concentrations of certain nutrients for plant growth, which negatively impact system’s productivity (Palm et al., 2018; Stathopoulou et al., 2021). Several studies have demonstrated system-induced nutrient deficiencies in coupled aquaponics which impede high crop yields (Bittsánszky et al., 2016; Ru et al., 2017; Tsoumalakou et al., 2022). Therefore, in intensive cultivations the reduced crop productivity considerably compromises the great advantage of aquaponics, *i.e.,* sustainability and low ecological footprint (Love et al., 2015).

During the last few years the new concept of decoupled aquaponics has been proposed that combines some of the afore-mentioned advantages of coupled aquaponics with solutions for the nutrient deficiency issues (Kloas et al., 2015; Delaide et al., 2016; Monsees et al., 2019). In this approach, aquaculture and hydroponics sub-systems are arranged in a multi-loop setup, as separate functional units that can be controlled independently (Delaide et al., 2016; Goddek et al., 2015). The water does not recirculate, but follows a unidirectional flow from fish tanks to hydroponic part, through an intermediate reservoir in which certain interventions on pH values and nutrient supplementation are performed. Consequently, the water physicochemical parameters are optimized to ensure high nutrient availability for plant growth, whereas the optimization measures do not affect the fish growth and welfare. Few empirical studies on the application of decoupled aquaponics and their comparison to conventional hydroponics have been published so far (Delaide et al., 2016; Al Tawaha et al., 2021; Rodgers et al., 2022). Their results confirm the efficacy of the new system in producing high yields, either comparable with hydroponics tomato yield (Suhl et al., 2016) and butterhead lettuce (Monsees et al., 2019), or outweighing hydroponics by a 39% increase in lettuce production according to Delaide et al. (2016). However, more works are needed in order to establish the positive contribution of decoupled systems in sustainable intensive crop production, and this would be served by thorough comparisons with hydroponics and coupled aquaponics performance.

The aquaponics-related literature, although extensive concerning coupled systems, has been severely limited to the study of crops’ growth responses, ignoring the functional ones (Buzby et al., 2016; Krastanova et al., 2022). Measuring only the yield or growth traits gives information on system’s productivity potential. Yet, it is not efficient to analyze the performance of a multi-component system like aquaponics, and describe its function when some variables change (Tsoumalakou et al., 2023a). Instead, incorporating the crop’s functional responses in the study elucidates plant fundamental processes that ultimately drive growth. Additionally, the assessment of functional responses allow identifying system constrains and proposing specific and effective optimization measures (Tsoumalakou et al., 2022). This connection between crop function, *i.e.*, physiology and productivity under aquaponics cultivation is an important gap in the relevant literature.

Sweet basil (*Ocinum basilicum* L.) is an aromatic herb with high commercial value because of its numerous uses (Avdouli et al., 2021). Except of culinary, ornamental and industrial use, basil has medicinal and especially antimicrobial properties that rely on crop quality and productivity (Scagel, Lee & Mitchell, 2019). Both quality and quantity of the produce depend on the cultivation conditions and systems. Basil is commercially cultivated in a wide range of growing systems including agricultural fields, hydroponic facilities. Basil has been regularly studied in aquaponic systems, because of its importance in fresh and processed markets, comparatively low nutrient demands, and high yield in short time periods, allowing successive harvests (Knaus et al., 2020; Rodgers et al., 2022).

The present study aims at bridging the above-mentioned research gaps related to (i) the connection between plant function and productivity in coupled and decoupled aquaponics, and (ii) a thorough comparison of the two distinct aquaponics system with the hydroponics one. To this target, we performed an experiment in a pilot-scale aquaponics greenhouse in which basil was co-cultivated with red tilapia and grown in both coupled and decoupled systems, with hydroponic systems serving as control. We examined crucial functional characteristics, scrutinizing processes that form the underlying mechanisms of growth performance. We focused on photosynthesis-related functional and biochemical parameters, such as gas exchange, the status and efficiency of the photosynthetic apparatus, and photosynthetic pigments content. All the above are linked to plant growth and productivity parameters along with plant nutrient status, in order to succeed an integrated approach of plant performance under the three different cultivation systems.

## Materials & Methods

### Aquaponics unit

**Figure 1:**
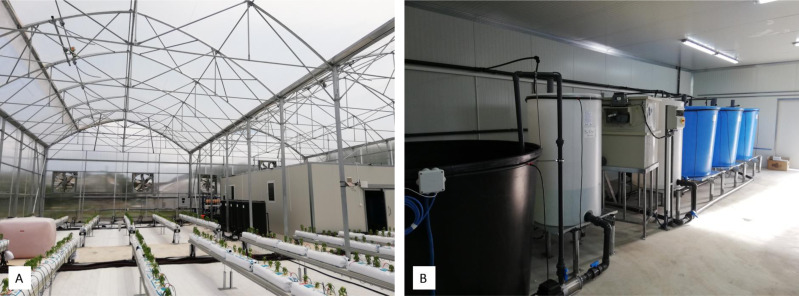
The aquaponics unit. Plant cultivation area (A) and RAS chamber (B) of the pilot-scale aquaponics greenhouse at the first days of the experiment.

**Figure 2:**
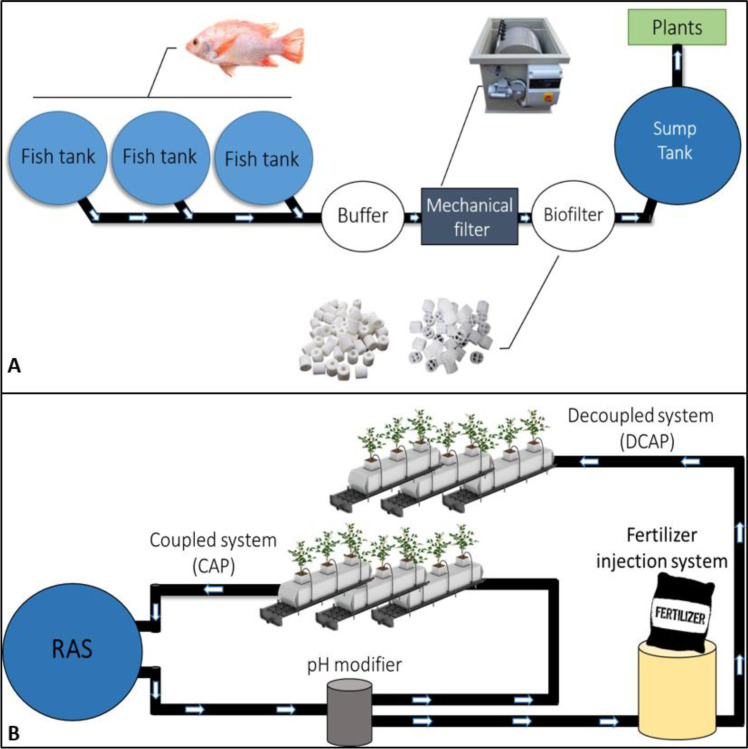
RAS and coupled/decoupled aquaponic systems. Scheme of the aquaponics system set up. (A), the RAS system and (B), coupled and decoupled systems.

A pilot-scale aquaponics unit has been installed and operating in the experimental greenhouse of the University of Thessaly, located in Velestino (Central Greece). The greenhouse consists of the plant cultivation area (360 m^2^) and a chamber, where the RAS is located (80 m^2^) (Fig. 1). The plant cultivation system in hydroponic setup consists of 18 channels. Each channel is 8.5 m long and bear 8 perlite slabs (Fig. 1A). The RAS (Fig. 1B) consists of (i) three large fish tanks of 1,500 L capacity (the blue tanks at the right side of Fig. 1B), (ii) an intermediate tank (buffer) of 650 L, (iii) a mechanical filter (Rotary Drum Filter, ProfIDrum B.V., Scandia, MN, USA), (iv) the biofilter, and (v) a sump tank of 2,500 L (the black one on the left side of Fig. 1B). The water flow among the sub-units of RAS is illustrated in Fig. 2A. The water flows unidirectionally from the fish tanks, through the buffer tank to the mechanical filter, where the solid fish waste is removed. Then it flows to the biofilter where the nitrifying bacteria perform the nitrification process in the ammonia-rich fish tanks-derived water. Finally, the water is collected to the sump tank and is directed to the plant cultivation area. The tanks are connected to each other with a system of pipes and valves to regulate the direction and rate of water flow.

The biofilter was made of ceramic rings (15 mm) and K1 (Kaldness media of 1 mm) that were colonized by nitrifying bacteria (Prodibio, Biodigest; Marseille, France). Prior to the experiment, a three-month period of biofilter set up was allowed for bacteria establishment and for effective rates of ammonia oxidation to nitrates to be obtained. The aeration of fish tanks and biological filters was supported by an air blower (Airtech Europe GmbH, Germany, 100 L h ^−1^) with twenty-two medium pore air diffusers (KW Airstone, 4 inch).

A greenhouse climate controller was used to manage the greenhouse microclimate. The system controlled the operation of the air heater, the vents and the pad and fan evaporative cooling system so that the greenhouse microclimate control settings could be obtained (temperature set points: heating 18 °C, ventilation 21 °C, evaporative cooling 26 °C, relative humidity set point: dehumidification 85%). The microclimate conditions (air temperature, relative humidity and solar radiation) inside the greenhouse were monitored every 1 min (by means of a temperature and humidity sensor and by a pyranometer) and the 10 min average values were recorded in a relevant data base.

### Experimental design and plant material

Basil seedlings (*Ocinum basilicum*, var. Genovese) of 6 cm height were transplanted at a density of 4 plants per slab, resulting in 2.4 plants/m^2^. The 18 channels were allocated to three treatments in a randomized complete block design (six channels per treatment, 192 plants per treatment). The treatments refer to three different nutrient solutions as follows:

•  hydroponic solution (HP): control

•  coupled aquaponic system (CAP): solution directly from the fish tanks

•  decoupled aquaponic system (DCAP): solution derived from fish, but enriched with nutrients by adding the necessary amounts of fertilizers to reach the target values of the HP.

The design of the two aquaponic treatments is illustrated in Fig. 2B, which depicts the flow of the water after leaving the above-described RAS (Fig. 2A). In the CAP treatment the water from RAS flows through the pH modifier to correct it to 5.6 (details below). Then it is directed to the channels of CAP basils. Closing the loop, the drainage solution of CAP plants is collected and returned to the fish tanks (after UV sterilization). The open loop at the right side of Fig. 2B depicts the solution flow in the DCAP treatment; the RAS water after the pH modifier is directed to the fertilizer injection system where the pre-determined fertilizer amounts are added and diluted (details below). Subsequently, the solution is distributed to the channels of DCAP basil. The drainage solution of this treatment is not re-circulated but discharged.

Mineral fertilizers were used to prepare the HP solution, according to the nutrient formula shown in Table 1. The same target values were used for the DCAP solution, in which the exact concentration of nutrients needed to be amended in the fish-derived water were determined after measuring its nutrient content on a weekly basis and modifying the input concentrations accordingly. The overall amount of fertilizers used for DCAP was reduced by 10% compared with HP treatment.

**Table 1:** Target nutrient concentrations in HP solution for basil.

**Macronutrients**	**Concentration (mg/L)**	**Micronutrients**	**Concentration (µmol/L)**
NO_3_^−^	682	Fe	5
NH_4_^+^	18	B	20
P	97	Cu	1
K	200	Mn	5
Ca	150	Zn	5
Mg	80	Mo	5
S	288		

The pH of all three treatment solutions was kept constant at 5.6 to ensure high nutrient availability to plants. The exact pH value was reached by adding a mix of nitric (65%), phosphoric (85%) and sulfuric acid (96%) for all three treatments in the fertilizer injection system. Specifically, nitric (1.07 L), phosphoric (1.06 L) and sulfuric acid (0.43 L) per 100 L nutrient solution were added.

Basil plants were watered with a drip irrigation system (2.31 L h ^−1^). The experiment lasted for 60 days (D) from May to July 2021, during which two harvests were performed, at the middle (D35) and the end (D60) of the growth period. In the first one the aerial part of all plants was cut and the plants were left to re-grow until the second, final harvest.

### Fish stocking

Red tilapias (*Oreochromis spp*.) were reproduced and reared for six months before the start of the experiment on the premises of the Aquaponic laboratory at the Department of Ichthyology and Aquatic Environment, University of Thessaly. The fish were acclimatized for 15 days and after this period, they were weighted and equally distributed at the three aquaponic tanks. The number of fish per aquaponic system was determined using the equation of the carrying capacity of an aquarium (Hirayama, 1974). The system was stocked with 410 red tilapias allocated according to their size to the three tanks, having a total of 22.56 kg fish biomass. Fish were fed three times per day (9:00, 13:00 and 17:00) *ad libitum* with Prodac Pondsticks Color (crude protein 27%). This feeding rate was kept constant throughout the experiment. At the end of the experiment fish were weighted again and final biomass was 50 kg. During the process of biomass determination, fish were anesthetized with Tricaine methanesulfonate (MS 222, 5 mg/L). The surviving animals were kept in the tanks to run the aquaponic system and to continue with the next experiment. The EU Directive 2010/63/EU concerning the protection and welfare of experimental animals was followed in all the steps of the experiment. FELASA-accredited scientists implemented all the animal-related experimental procedures (functions A–D).The experimental protocol was approved by the Animal Care and Use Ethics Committee (approval number 6/28-01-2021) and conducted at the registered experimental facility (EL-43BIO/exp-02) at the University of Thessaly.

### Plant measurements

Several plant functional parameters were measured at 15-days intervals throughout the growth period, such as photosynthetic pigments content, gas exchange, chlorophyll a fluorescence and Photochemical Reflectance Index (PRI). As mentioned before, two harvests were performed (D35 and D60), in both of which, plant growth parameters along with the leaf nutrient status were assessed.

### Concentration of photosynthetic pigments

The chlorophyll and carotenoid content of fresh leaves were determined at two leaves per plant and 10 plants per treatment, according to Lichtenthaler & Wellburn (1983). Briefly, leaf discs (0.64 cm^2^) were taken with a cork borer and extracted in a mortar with acetone 80% v/v. After centrifugation at 4,000 rpm for 10 min, the absorbance of the supernatant was read at 720, 663, 646 and 470nm using Shimadzu’s UV-1900 dual-beam spectrophotometer. The data were used in the relevant equations for this particular solvent and the concentration of chlorophyll a (chla), chlorophyll b (chlb), carotenoids (car) and the ratio of carotenoids to chlorophylls (car/chls) were expressed in µg cm^−2^.

### Leaf gas exchange and light response curves

Gas exchange measurements were performed with a portable photosynthesis system (LI-6400/XT, LI-COR, Lincoln, NE, USA) during clear days, between 9:30 and 11:30 in the morning. Both instantaneous gas exchange measurements, and light response curves (LCs) were assessed on different days. The leaf chamber conditions were set in all cases at 400 ppm CO_2_ (6400-01 CO_2_ Injector) and 25  °C to represent the ambient environmental conditions yet avoiding fluctuations that could affect the comparisons among treatments. Net photosynthetic rate (A_n_, µmol m^−2^ s ^−1^), transpiration rate (Tr, mmol m^−2^ s ^−1^), and stomatal conductance (gs, mol m^−2^ s^−1^) were determined, while water use efficiency (WUE, µmol mmol^−1^) was estimated as An-to-Tr ratio. The instantaneous gas exchange measurements were performed on thirty replicates per treatment (one mature leaf per plant) at 15-days intervals, under 500 µmol m^−2^ s ^−1^ achieved with the LED lamp of the system. This light intensity corresponded to the ambient light environment inside the greenhouse, and we preferably used external lamp to avoid light fluctuations during measurements. The LCs were performed twice in the growth period (days 30 and 55) on ten replicates per treatment. The light intensities used in LCs were 2,000, 1,500, 1,200, 800, 400, 200, 100, 80, 40, 20 and 0 µmol m^−2^ s^−1^, the duration of each step being at least 3 min. According to Markos & Kyparissis (2011), this high-to-zero light protocol prevents stomatal closure and re-opening. In light curves we used the modified non-rectangular hyperbola proposed by the above-mentioned authors to assess maximum photosynthetic rate (Amax) and quantum yield of photosynthesis (a), the latter referring to moles of CO_2_ per mole PAR (photosynthetically active radiation) incident on leaf surface.

### *In vivo* chlorophyll a fluorescence

All fluorescence measurements were performed with the Handy PEA+ fluorimeter (Plant Efficiency Analyser, Hansatech Instruments, Ltd., Norfolk, UK), at predawn (thus fully dark-adapted state), at thirty replicates per treatment (one mature leaf per plant). The conditions for obtaining the chlorophyll a fluorescence transients were the following: two seconds illumination with 3,000 µmol photons m^−2^ s^−1^, provided by a red LED array and centered at 650 nm. PeaPlus Software v.1-13 (Hansatech Instruments Ltd, Pentney, UK) was used for analyzing the OJIP transients. The parameters selected from the JIP test (Strasser, Tsimilli-Michael & Srivastava, 2004) are summarized in Table 2, along with their physiological meaning.

**Table 2:** Chlorophyll fluorescence parameters derived from the fast OJIP fluorescence induction.

**Fluorescence parameters**	
*F*_M_	Maximal fluorescence from a dark-adapted leaf
*F*_V_	Maximal variable fluorescence from a dark-adapted leaf. FV=FM −F0
*F*_V_/ *F*_M_	Maximum quantum efficiency of PSII photochemistry
*φ*Eo	Quantum yield for electron transport at *t* = 0
*φ*Ro	Quantum yield of electron transport flux from Q to the final PSI acceptors
*ψ*Eo	Probability that a trapped exciton moves an electron into the electron transport chain beyond
*δ*Ro	Efficiency/probability with which an electron from QB is transferred until PSI acceptors
Vi	Relative variable fluorescence at phase I of the fluorescence induction curve
1-Vi	Measure of relative amplitude of the IP phase in OJIP transient, related to the size of the pools of final PSI electron acceptors
1/Vi	Relative measure of the pool size of final electron acceptors of PSI
ABS/RC	Absorption flux (for PSII antenna chls) per reaction center (RC)
TR_0_/RC	Trapped energy flux per RC (at *t* = 0)
DI_0_/RC	Dissipated energy flux per RC (at *t* = 0)
PI_TOTAL_	Performance index total for energy conservation from photons absorbed by PSII to the reduction of PSI end acceptors
PI_ABS_	Performance index for energy conservation from photons absorbed by PSII antenna
Sm	Normalized area above the OJIP curve

### PRI

PRI was measured with the handheld reflectance meter PlantPen PRI120 (Photon Systems Instruments, Czech Republic). The instrument measures leaf reflectance (R) in two narrow wavelength bands centered close to 531 nm and 570 nm and its output is the value of PRI calculated according to Gamon, Peñuelas & Field (1992) as follows:

\begin{eqnarray*}PRI= \frac{R531-R570}{R531+R570} \end{eqnarray*}

PRI measurements were carried out at two time-points during the day on the same leaves; the first at predawn and the second at midday, on thirty replicates per treatment (one mature leaf per plant).

### Growth parameters

In the initial harvest on day 35, the aerial part of all plants was cut and 10 plants per treatment were sampled for biomass determination. For this measurement the whole plant was removed, including the roots. Special care was taken to remove the whole root from the perlite of the slags. Afterwards, leaves, stems and roots were separately collected, and the samples were oven-dried (80 °C for 48 h) to estimate the dry weight (DW) of each plant part and calculate the root-to-shoot ratio. The same procedure was repeated in the final harvest where all the experimental plants were removed.

### Nutrient concentration in basil leaves

Dry leaf tissue from initial and final harvests was used to conduct the nutrient elemental analysis for macronutrient (N, P, K, Ca, Mg expressed as % DW) and micronutrient (Fe, Zn, Mn, Cu expressed as ppm DW). The concentrations of nutrients were determined by ICP-OES Spectrophotometer (SPECTRO Analytical Instruments GmbH, Kleve, Germany). Three samples per treatment were analyzed (0.25 g each). The samples were digested for two hours at 30 °C with 4.4 mL of the digestion solution, according to slightly modified Kjeldahl method (1.94 mL concentrated sulfuric acid, 2.82 mg Se, 82.13 mg Li_2_SO _4_, and 1.94 mL 30% H_2_O_2_). Afterwards, samples were left to reach room temperature, diluted up to 50 mL with distilled water and then were subjected to the elemental analysis (Avdouli et al., 2021).

### Statistical analysis

All data were examined for statistical significant differences among treatments using one-way ANOVA, followed by Tukey’s *post-hoc* tests (*p* ≤ 0.05). In the cases of root/shoot and car/chl ratios, WUE and the chlorophyll fluorescence parameters where the assumptions of ANOVA were not met, the non-parametric test Kruskal–Wallis was used. All the analyses were performed with the free statistical software Jasp 0.14.0.0 (JASP Team 2021 Computer Software).

## Results

### Growth parameters

**Figure 3:**
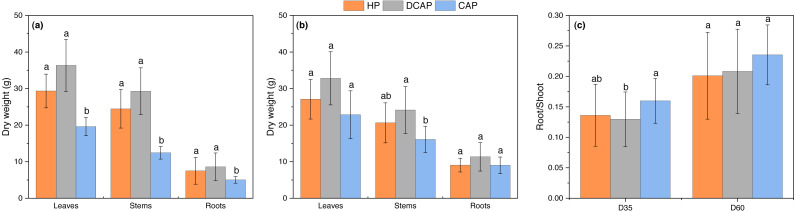
Basil growth parameters. Dry weight of basil leaves, stems and roots at (A) D35 and (B) D60 and (C) the root-to-shoot ratio for both harvests (mean ±SD). Different letters in each plant part denote statistically significant differences among treatments (*p* < 0.05).

The biomass allocation pattern in various plant parts at the initial (D35) and final (D60) harvest is shown in Fig. 3. In all cases, DCAP outweighed HP and CAP. Notably, in the initial harvest leaves of DCAP reached 36.34 ± 7.12 g of dry weight, which was higher but not significantly different from HP (29.35 ± 4.59 g), yet statistically significant differences (*p* < 0.001) appeared with CAP (19.61 ± 2.49 g). Exactly the same picture was displayed in the dry weight of stems (DCAP = 29.27 ± 6.39 g, HP = 24.47 ± 5.27 g and CAP = 12.43 ± 1.73 g, the latter being significantly lower than DCAP at p<0.001), and roots (DCAP = 8.60 ± 3.77 g, HP = 7.49 ± 3.44 g and CAP = 5.03 ± 0.91 g, being significantly lower than DCAP at *p* = 0.006). As shown in Fig. 3, the trend for higher values in DCAP compared with HP was not significant in any plant part. At the final harvest, leaves and roots showed no significant differences among treatments (ranging from 22.84 g to 32.81 g in leaves and 9.01 g to 11.33 g in roots). However, the stems of CAP plants were lower than DCAP at *p* = 0.009 (16.10 ± 3.60 g in CAP, 24.11 ± 6.43 g in DCAP and 20.63 ± 5.46 g in HP). Concerning the between-harvests differences, noteworthy is the increase in biomass accumulation in leaves, stems and roots of CAP plants (14.1%, 22.8% and 44.2% increase, respectively), in contrast with DCAP and HP which maintained similar leaf weights but slightly decreased the stem weights (2.11 % decrease for DCAP and 0.62% HP). Figure 3C illustrates the partitioning of biomass to shoot and root, through their ratio. CAP plants exhibited the highest value of the ratio (0.16 ± 0.04) denoting that they allocated a greater proportion of their biomass to the root system, while the DCAP and HP plants showed lower ratios (0.13 ± 0.05 and 0.14 ± 0.05 respectively), with the DCAP ratio being significantly different from CAP (*p* = 0.034).

### Photosynthetic pigments content

**Figure 4:**
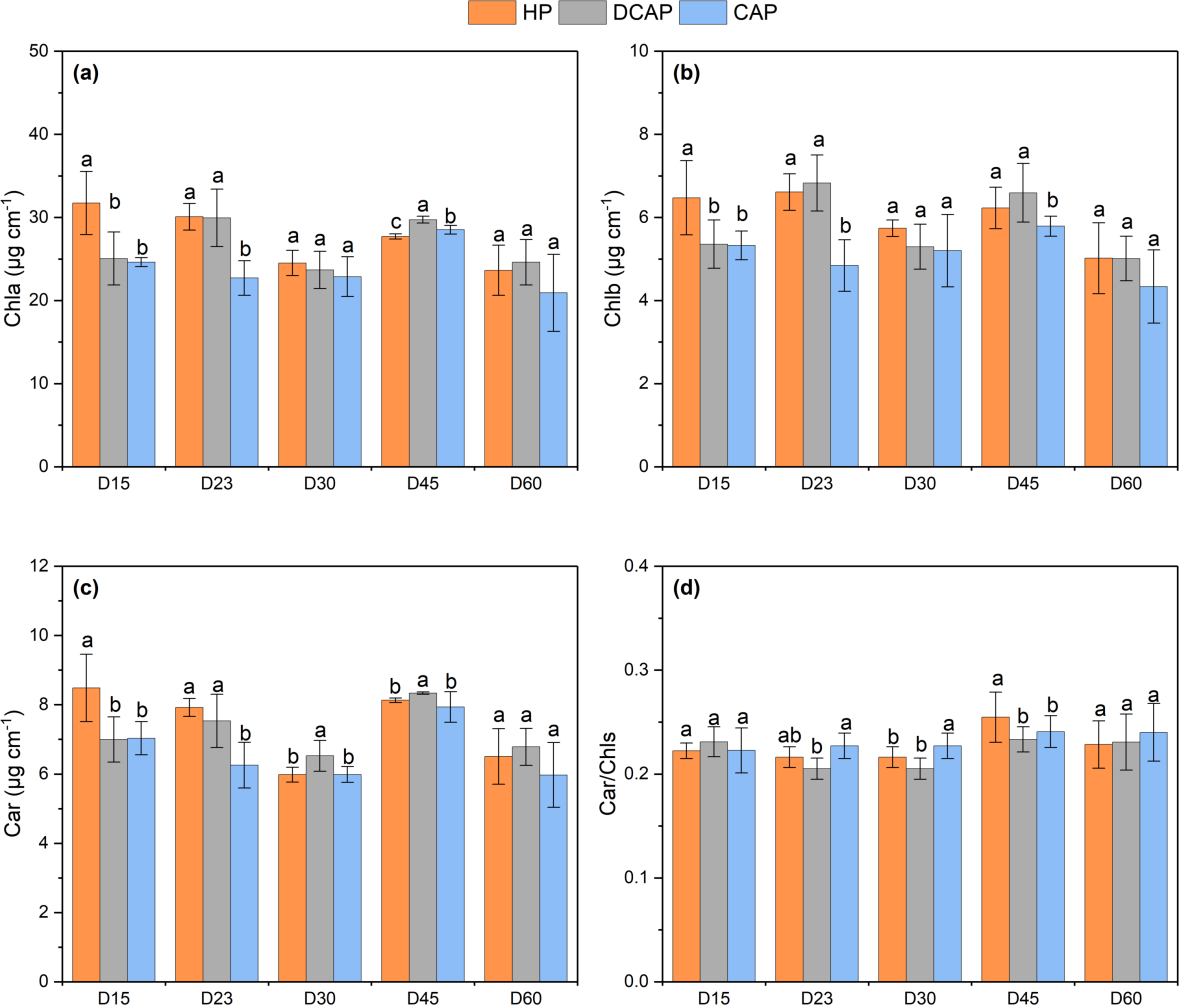
Photosynthetic pigments content. Concentration of photosynthetic pigments (mean ± SD) in basil leaves during the experimental period: (A) Chla; (B) Chlb; (C) Car; (D) Car/Chls. Different letters denote statistically significant differences among treatments at each experimental day (*p* ≤ 0.05). Chla, Chlorophylla; Chlb, Chlorophyllb; Car, Carotenoids; Car/Chls, the ratio of Carotenoids to total Chlorophylls.

The photosynthetic pigments content (Fig. 4) was always higher in DCAP leaves, except of the first measurement on day 15, where HP outweigh the other treatments (on D15: 31.73 ± 3.79 µg of chla, 6.47 ± 0.89 µg chlb and 8.48 ± 0.97 µg car for HP and respectively 25.06 ± 3.19, 5.35 ± 0.58 and 7.00 ± 0.65 µg for DCAP and 24.61 ± 0.53 µg of chla, 5.33 ± 0.34 µg of chlb 7.03 ± 0.48 µg for car for CAP, all expressed per cm^2^). The fluctuation of chla, chlb and car during the cultivation period followed a similar pattern with slightly lower values at the maturity stage, *i.e.*, before harvests (at D30 and D60) compared with the other time-points. The before-harvests measurements were also characterized by the absence of statistically significant differences among treatments unlike the days before and after them. Using the concentration of chla as a representative example, at D30 the values of all treatments were similar (ranging from 22.87 to 24.51 µg cm^−2^) and the same also holds for D60 (ranging from 20.92 to .23.63 µg cm^−2^). However, on D23, DCAP and HP showed increased values (29.94 ± 3.45 and 30.08 ± 1.59 µg cm^−2^ respectively) than CAP (22.71 µg cm^−2^), a difference that was significant at *p* <0.001. Also at D45, statistically significant differences were recorded between DCAP and HP (chla content of 29.73 ± 0.41 and 27.70 ± 0.31 µg cm ^−2^respectively, *p* <0.001), between DCAP and CAP (the latter of 28.52 ± 0.52 µg cm^−2^, *p* = 0.0011) and also between HP and CAP (*p* = 0.011). The general picture that emerged was that CAP plants exhibited constantly decreased pigment contents in comparison with HP and DCAP, which in most cases showed similar values. The car/chl ratio was significantly decreased in DCAP leaves in the middle three out of five time-points of pigment determination. The DCAP ratio on D23 was 0.22 ± 0.01, different from CAP (0.23 ± 0.01) at *p* < 0.001; at D30 was 0.21 ± 0.01 µg cm^−2^, different from CAP (0.22 ± 0.02) at *p* = 0.009; on D45 it reached 0.23 ± 0.01 µg cm^−2^, different from HP (0.25 ± 0.02) at *p* = 0.023).

### Gas exchange and light response curves

**Figure 5:**
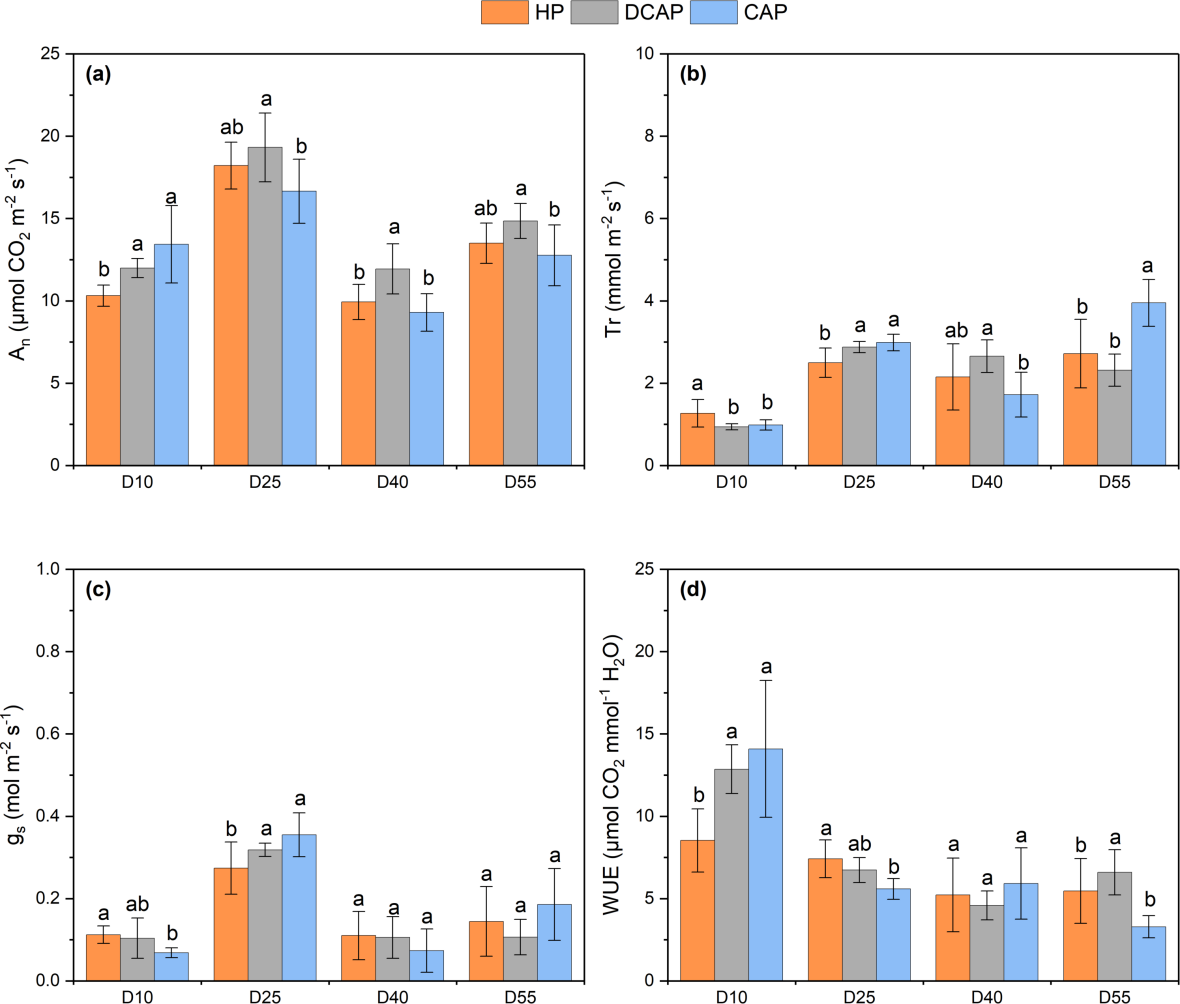
Gas exchange parameters. Gas exchange parameters during the experimental period: (A) net photosynthetic rate; (B) transpiration rate; (C) stomatal conductance; (D) water use efficiency (mean ± SD). Different letters denote statistically significant differences among treatments at each experimental day (*p* ≤ 0.05). An, rate of net photosynthesis; Tr, transpiration rate; gs, stomatal conductance; WUE, instantaneous water use efficiency.

Instantaneous gas exchange measurements were performed throughout the cultivation period as shown in Fig. 5. The profile of change in all measured parameters indicate the two phases of the photosynthetic machinery maturity, during the first half of the experiment which ended with the initial harvest (D35), and the second half in which the aerial part was regrown until the final harvest. This maturation process was reflected in the lower value of gas exchange parameters at D10 compared to a more mature photosynthetic machinery at D25, as well as in the second half the differences between D40 and D55. Taking net CO_2_ assimilation rate (An) as a representative parameter, the increase between D10 and D25 reached 43.4% for HP, 37.9% for DCAP and 19.3% for CAP, while lower percentage of increase was recorded between D40 and D55 (19.6% for HP, 15.1% for DCAP and 20.8% for CAP). An was higher in DCAP leaves, except of the first measurement of D10. The highest value of An was reached on D25 for all treatments (18.22 ± 1.42 for HP, 19.32 ±2.09 for DCAP and 16.66 ± 1.95 for CAP, all expressed in µmol CO_2_ m^−2^ s^−1^), with the difference between DCAP and CAP being statistically significant (*p* = 0.037). The higher An of DCAP plants cannot be attributed to higher stomatal conductance Fig. 5C, since the gs differences among treatments were minimal at all time-points (ranging from 10.3% decrease in CAP compared with the other two groups on D10, *p* = 0.006, to 22.7% decrease in HP compared with the other two groups on D25, *p* = 0.049). This was also the case in Tr throughout the cultivation period, with the most pronounced difference appearing between DCAP and CAP at the last measurement (2.72 ± 0.83 mmol m^−2^ s^−1^ in DCAP and 3.95 ± 0.57 mmol m^−2^ s ^−1^ in CAP, *p* < 0.001). High WUE of DCAP was evident in most measurements, while CAP showed significantly decreased values on D25 and D55 (on D25 HP was 7.42 ± 1.15, DCAP 6.57 ± 0.88 and CAP 5.66 ± 2.22 at *p* = 0.009 and on D55 HP was 4.94 ± 1.81, DCAP 6.92 ± 1.98 and CAP 4.10 ± 2.22 at *p* = 0.027, all values expressed as µmol CO_2_ mmol^−1^ H_2_O). At the first days of the experiment significantly lower A_n_ of HP leaves (10.31 ± 0.64 µmol CO_2_ m ^−2^ s^−1^ on D10, *p* = 0.028) combined with higher Tr (1.27 ± 0.33 mmol m^−2^ s ^−1^ on D10, *p* = 0.027) resulted in lower value of WUE (8.53 ± 1.92 µmol CO_2_ mmol^−1^ H_2_O, *p* = 0.006) compared to the other two treatments, a difference that was evened out in the subsequent measurements.

**Figure 6:**
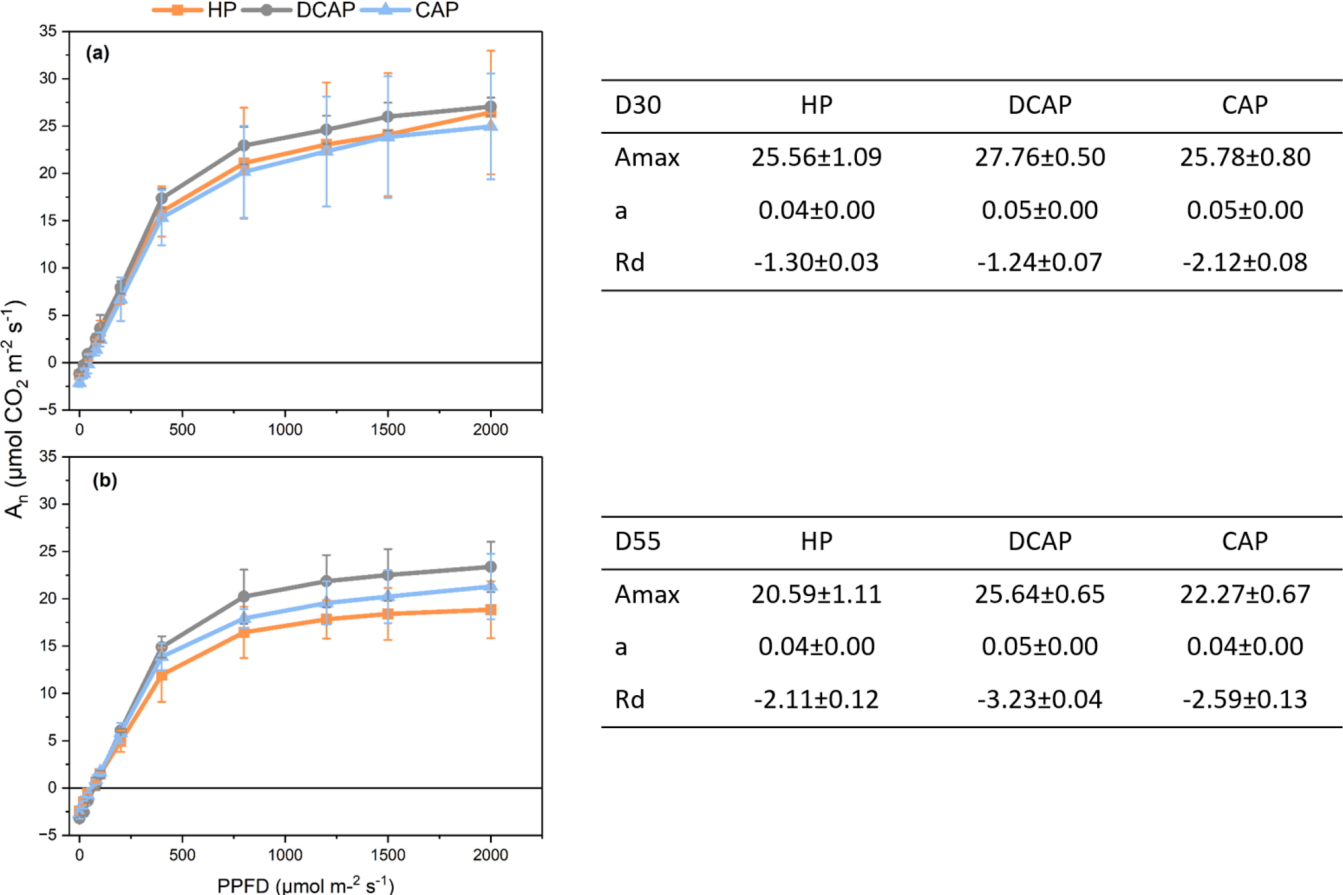
Photosynthetic light response curves. Light response curves at (A) D30 and (B) D55 (mean ± SD). The values of Amax, a, and Rd are presented in the tables next to each layer, where the absence of statistical signs denotes no statistically significant differences among treatments (*p* ≤ 0.05). n, rate of net photosynthesis; Amax, photosynthetic capacity; a, quantum yield of photosynthesis; Rd, dark respiration rate.

The photosynthetic light response curves were performed on D30 and D55 (Fig. 6). Important parameters, like photosynthetic capacity (Amax), the quantum yield of photosynthesis (a) and dark respiration rate (Rd) were derived from LCs. DCAP plants showed a superior performance in terms of Amax and a, compared with the other two treatments at both time-points (Amax on D30 was 27.76 ± 0.50 for DCAP, 25.56 ± 1.09 for HP and 25.78 ± 0.80 for CAP and on D55 was 25.64 ± 0.65, 20.59 ± 1.11 and 22.27 ± 0.67 respectively; a reached 0.04 ± 0,001 in DCAP, 0.05 ±0.002 in HP and 0.05 ± 0.002 in CAP on D30 and 0.04 ±0.002, 0.05 ± 0.001 and 0.04 ± 0.002 respectively on D55, all values expressed as µmol CO_2_ m ^−2^ s^−1^). Nevertheless, none of the above-mentioned differences were statistically significant. It should be highlighted here that in the first measurement CAP plants exhibited similar to HP values of photosynthesis along light gradient but in the final measurement held an intermediate position between DCAP and HP, with the latter exhibiting the lowest values (Fig. 6). Rd was comparable in all treatments at Day 35, with a trend for higher values in CAP (−2.12 ± 0.08 *versus* −1.30 ± 0.03 in HP and −1.24 ± 0.07 in DCAP, all in µmol CO_2_ m^−2^ s^−1^), however, at the end of the experiment DCAP showed increased dark respiration compared with HP (−3.23 ± 0.04 and −2.11 respectively, all in µmol CO_2_ m^−2^ s ^−1^), a difference that was marginally non-significant.

### Chlorophyll a *in vivo* fluorescence

**Figure 7:**
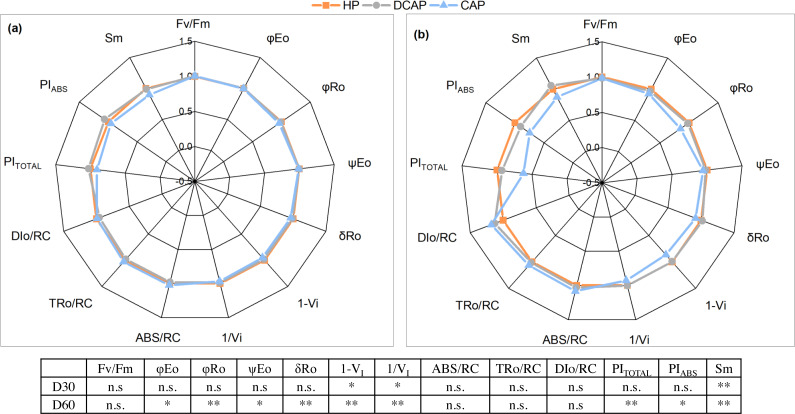
Chlorophyll a fluorescence parameters. Radar plots representing the changes in the values of chlorophyll a fluorescence parameters from the JIP-test, for days 30 (A) and 60 (B) after transplantation. The values are normalized to HP values (regarded as 1.0). The results of statistical analyses are presented in the bottom table, where (n.s. means non-significant differences among treatments, the single asterisk (*) indicates differences between CAP and HP treatments, double asterisk indicates differences between CAP and both HP and DCAP treatments (*p* ≤ 0.05). Explanation of the measured parameters in Table 2.

Radar plots in Fig. 7 depict the changes of *in vivo* chlorophyll a fluorescence of DCAP and CAP in comparison with HP (control), *i.e.*, the values of DCAP and CAP are normalized to the values of HP; these normalized values are presented hereinafter (the actual fluorescence values may be found in the Supplementary Material). The parameters presented in Fig. 7 are explained in Table 2. No significant differences between HP and DCAP were recorded in any parameter or experimental date. The most significant changes in OJIP parameters were observed on D60. PI_Total_ and PI_ABS_ which are performance indices of total photosynthetic efficiency and conservation of photons absorbed by the PSII antenna respectively, were significantly decreased under CAP treatment (CAP = 0.621 differing at *p* < 0.001 with 0.930 in DCAP and 1 in HP for PI_Total_; PI_ABS_ was 0.621, 0.902 and 1 respectively, *p* = 0.016). Similarly, a significant decrease was evident in CAP in functional parameters related to the quantum yield of electron transport to intermediate (*φ*Eo) and final (*φ*Ro) acceptors (0.927, 0.967 and 1 in CAP, DCAP and HP for *φ*Eo, *p* = 0.03; 0.851, 0.979 and 1, *p* ≤ 0.001 for *φ*Ro respectively) as well as the efficiency of these two parts of transport in the photosynthetic chain, *i.e.*, ψEo (0.941 in CAP, 0.980 in DCAP and 1 in HP, *p* = 0.010) and δRo 0.919 in CAP, 1.019 in DCAP and 1 in HP, *p* = 0.003 compared to DCAP and *p* <0.001 compared to HP). These results may be related to the diminished relative pool size of total electron carriers reflected in Sm of CAP plants in both measurement dates (0.874 compared with 1.058 in DCAP, *p* = 0.005; also compared with 1 in HP, *p* = 0.001). In addition, the PSI-related events such as the content of PSI reaction centers (1-V_I_) and relative size of the pools of final PSI electron acceptors depicted in 1/V_I_ showed early signs of PSI limitations under CAP, already from D30 (1-V_I_ was 0.865, 0.996 and 1, in CAP, DCAP and HP respectively, *p* <0.001; the respective values of 1/V_I_ were 0.928, 1.002, 1, with *p* = 0.003 when CAP was compared to DCAP and *p* < 0.001 compared to HP). On the contrary, Fv/Fm, and the fluxes of energy (absorbed, ABS/RC; trapped, TRo/RC; dissipated, DIo/RC) remained unaffected by CAP treatment.

### PRI

**Figure 8:**
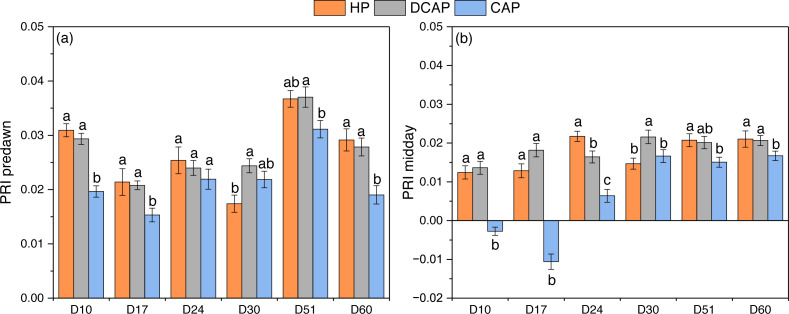
PRI index. PRI index of basil leaves measured at (A) predawn and (B) midday (mean ± SD). Different letters indicate significant differences among treatments at each experimental day (*p* ≤ 0.05).

Figure 8 illustrates the results of PRI index based on leaf reflectance measurements at predawn and noon. At predawn measurements, HP and DCAP treatments produced similar PRI values, ranging from 0.021 on D17 to 0.037 on D51. Both treatments significantly outperformed CAP in all time-points except D24 (CAP value on D17 was 0.015 ± 0.001, differing at *p* = 0.001 and *p* = 0.024 from DCAP and HP respectively; CAP value on D51 was 0.031 ± 0.002 significantly different from DCAP at *p* = 0.038). At midday measurements, DCAP and HP showed slight fluctuations (0.014 to 0.022 for DCAP and 0.012 to 0.022 for HP), being significantly different only on D24 (*p* = 0.041) and D30 (*p* = 0.002). The midday PRI was, as expected, lower than the pre-dawn with CAP plants reaching negative values at the first two dates (−0.003 ± 0.001 on D10, *p* < 0.001 and −0.011 ±0.002 on D17, *p* < 0.001, the *p* values referring to differences with both DCAP and HP). The pre-dawn PRI of HP and DCAP showed a small decline between D17 and D30, before the initial harvest, but recovered to high values after that (52.6% and 34.2% increase between D30 and D51 for HP and DCAP, respectively).

### Leaf elemental analysis

**Figure 9:**
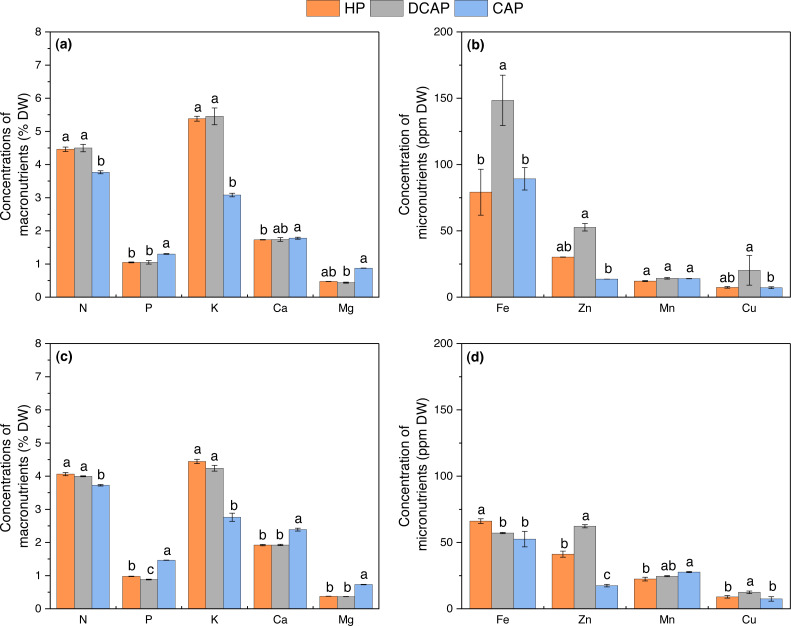
Leaf nutrients concentration. The nutrient composition of basil leaves at the initial harvest at D35 ((A) macronutrients; (B) micronutrients) and the final one at D60 ((C) macronutrients; (D) micronutrients) (mean ± SD). Different letters indicate significant differences among treatments for each element and harvest day (*p* ≤ 0.05).

The nutrient content of basil leaves was assessed after the two harvests, at the middle and end of the experiment (Fig. 9). The differences among treatments found in the first measurement (Figs. 9A, 9B) were also retained in the final one (Figs. 9C, 9D). There was not a clear picture on the direction of these differences, as CAP leaves compared to HP and DCAP contained significantly lower concentrations of N and K (3.77 ± 0.05, 4.46 ± 0.07 and 4.50 ± 0.11for N respectively, *p* < 0.001; 3.08 ± 0.05, 5.38 ± 0.07 and 5.45 ± 0.25 for K respectively, differences between CAP and HP at *p* = 0.037 and between CAP-DCAP at *p* = 0.013), yet higher P, Mg and Ca only in the final harvest 1.46 ± 0.003, 0.98 ± 0.01, 0.88 ±0.01 for P respectively, *p* < 0.001; 0.73 ± 0.01, 0.38 ± 0.003 and 0.36 ± 0.004 for Mg, *p* < 0.001; 2.39 ± 0.04, 1.92 ± 0.02, 1.92 ± 0.02 for Ca, *p* < 0.001). The macronutrients content of HP and DCAP were found to be similar, with the only exception being the relatively higher P of HP at the final harvest (0.98 ± 0.01 compared to 0.88 ± 0.1 in DCAP, *p* < 0.001). Concerning micronutrients, DCAP plants exhibited remarkably higher levels of Fe, Zn and Cu, compared with the other two treatments in the first harvest (148.43 ± 18.98 in DCAP, 79.15 ± 17.32 in HP (*p* = 0.004), 89.24 ± 8.42 in CAP (*p* = 0.008) for Fe; 52.67 ± 2.84, 30.19 ± 0.15 and 13.63 ± 0.03 for Zn respectively, *p* = 0.004; 12.42 ± 0.94, 8.97 ± 0.95, and 7.40 ± 1.64 for Cu respectively, *p* = 0.009). Although the differences in Zn and Cu were maintained in the final harvest (62.23 ± 1.21 DCAP, 41.20 ± 2.25 HP and 17.44 ± 0.94 CAP for Zn, *p* < 0.001; 12.42 ± 0.94, 8.97 ± 0.95 (*p* = 0.031), and 7.40 ± 1.64 (*p* = 0.006) respectively for Cu), they were reversed in the case of Fe, with HP outperforming DCAP and CAP (66.10 ± 1.78, 57.15 ± 0.46 and 52.54 ± 5.83 respectively, *p* = 0.038).

## Discussion

The continuous improvement of the aquaponic systems towards high productivity of both crops and fish, entails the incorporation of technical advancements but more importantly new approaches that might eliminate the bottlenecks of the system. Decoupled aquaponics have been introduced as a means to tackle the latter. In the present study a thorough investigation of both growth and functional responses of basil plants cultivated in DCAP, CAP and HP systems attempted to fill the gap of the relevant aquaponics research in plant function-related information. Indeed, although numerous studies have dealt so far with the productivity efficiency of aquaponics, the underlying mechanisms of plant physiology leading to this growth response has only recently begun to be investigated (Tsoumalakou et al., 2022; Tsoumalakou et al., 2023a; Tsoumalakou et al., 2023b).

### Growth response in the middle and end of the cultivation period

Basil growth as depicted in the accumulation of dry biomass was significantly higher in DCAP treatment compared with CAP, yet without being statistically different from HP (Fig. 3). Particularly in the initial harvest, DCAP leaves were 45% heavier compared with CAP and showed an insignificant 22% increase compared to HP leaves, with the same trends also appearing in stem and root biomass. Opposite results were reported by Rodgers et al. (2022) with hydroponic basil plants outperforming both coupled and decoupled aquaponic solution-treated plants. However, no direct comparisons are possible with our results due to shorter duration of their experiment (21 days) and more importantly, different experimental design; they complemented the decoupled system with a strict 25% of the chemical fertilizer used in their HP treatment, instead of continuous monitoring of nutrient content of the RAS water and the subsequent replenishment towards the HP target, as in our case. Monsees et al. (2019) working with lettuce as well as Delaide et al. (2021) and Suhl et al. (2016) with tomato followed our approach in fertilizer inputs of decoupled system, *i.e.*, a continuous assessment of the fish water nutrient load followed by the required additions of mineral fertilizer to reach the target concentrations of HP treatment. Collectively, they found similar growth responses and yields between decoupled aquaponics and hydroponics for both crops, corroborating our results. Interestingly, in a previous study with lettuce Delaide et al. (2016) reported a 40% increased growth in complemented aquaponics in comparison with conventional hydroponics.

### Photosynthetic pigments content and light use efficiency

DCAP treatment favored the photosynthetic pigments content of basil leaves, though HP resulted in virtually similar values at many time-points throughout the cultivation period (Fig. 4). CAP leaves contained significantly decreased photosynthetic pigments compared to the other two treatments. The inferiority of coupled aquaponics-treated plants in terms of chlorophyll content has been regularly reported in the relevant literature (Roosta, 2014; Rodgers et al., 2022). It is typically ascribed to the lower leaf elemental content of the coupled aquaponics treatments, especially to the sub-sufficient levels of N, which was also the case in the present study. It is widely recognized that leaf nitrogen content is directly correlated with chlorophyll concentration, with several species showing strong linear relationships (Evans, 1989). Moreover, the lower Fe content of CAP leaves may also explain the decreased chlorophyll content, as suggested by Roosta (2014), who attributed the chlorosis observed in aquaponic basil to Fe shortage. Likewise, aquaponics-grown spinach showed extensive chlorosis already from the 10 th day of cultivation in a CAP-like system, which was correlated with Fe-deficiency (Tsoumalakou et al., 2023b). Collectively, except of N, Fe is also a determinant of chlorophyll levels, since it is a co-factor of many enzymes involved in chlorophyll biosynthesis (Kasozi et al., 2019). Concerning DCAP and HP comparison, Rodgers et al. (2022) found no difference, whereas Suhl et al. (2016) noted that decoupled system considerably increased chlorophyll content. It should be highlighted here that in our experiment the car/chls ratio was lower in DCAP leaves, denoting a reduced need for photoprotection. In support of this finding, the PRI index of leaf reflectance was high at pre-dawn and midday measurements in both DCAP and HP plants (Fig. 8). PRI was originally developed to track the xanthophyll cycle pigment changes and whereby the light use efficiency (Levizou & Manetas, 2007; Gamon & Berry, 2012; Vanikiotis, Stagakis & Kyparissis, 2021), but has also been related to carotenoid to chlorophyll ratio in green leaves (Sims & Gamon, 2002). Notably, PRI variation over the diurnal cycle is primarily considered a function of xanthophyll cycle changes in response to stress, whilst long-term changes in PRI (during weeks or months) mainly reflect modifications of car-to-chl ratio as an acclimation to light environment or stress (Sims & Gamon, 2002). In the present work, DCAP and HP leaves showed constantly higher PRI, thus higher photosynthetic light use efficiency compared to CAP leaves, both in the short- and the long-term. The diurnal decline of PRI in CAP leaves implies a stress response.

### Photosynthetic performance

DCAP stimulated the photosynthetic performance of basil leaves throughout the experiment. The net CO_2_ assimilation rate was higher in DCAP leaves, with HP-treated leaves following and showing similar values with CAP (Fig. 5A. The superiority of DCAP photosynthesis cannot be attributed to higher stomatal conductance which showed minimal differences among treatments at all time-points (Fig. 5B), likewise transpiration rate and instantaneous WUE (Figs. 5C, 5D). The limited stomatal opening was reported to be the reason of reduced photosynthesis in basil grown under coupled aquaponics in comparison to hydroponics (Roosta, 2014) and this effect was ascribed to the influence of K deficiency. However, in the present study an interesting connection may arise between gas exchange and the state and efficiency of the photosynthetic machinery as revealed by light response curves and the monitoring of chlorophyll a *in vivo* fluorescence. LCs indicated that the machinery of DCAP leaves showed constantly higher photosynthetic capacity and more efficient use of light in the first linear part of the curve (Fig. 6). Fluorescence kinetics exhibited similar results, since sensitive indicators confirmed the high efficiency of PSII photochemistry in DCAP leaves. On the contrary, in the CAP leaves occurred a down-regulation of PSII activity as reflected in the significant decreases of quantum yield and efficiency of electron transport and probably related to reduced relative pools of total electron carriers. Additionally, early signs of PSI limitation were obvious in CAP plants, which may partly account for the overall inferior photosynthetic performance of this group. The poor nutritional state of CAP, notably the low N, K and Fe levels may explain the above-described decreased efficiency of the photosynthetic machinery. Several studies have established a cause-and-effect relationship between nutrients deficiency and a sub-optimal photosynthetic apparatus development which results in reductions of PSII and PSI photochemistry (Kalaji et al., 2018; Roosta, Estaji & Niknam, 2018). According to Samborska-Skutnik et al. (2020), Fe deficiency was responsible for the disruption of light absorption and the decreased activity of the primary quinone acceptor (QA), entailing impaired PSII function of radish. The findings of Kalaji et al. (2018) corroborate these results since it they confirm that nutrient deficiency negatively impact PSII function in two directions, *i.e.*, decreasing the efficiency of energy capture and the electron transport quantum yield. Finally, in the experiment of Roosta (2014), basil cultivated in coupled aquaponics exhibited a remarkably reduced maximum yield of PSII photochemistry compared to hydroponics plants, which was connected to K, Fe and Mn deficiencies.

### Nutritional state of leaves

The nutritional status of basil leaves was similar for HP and DCAP treatments in most macronutrients, yet remarkable increases in Fe, Zn and Cu content in DCAP leaves were evident (Fig. 9). Despite the same target values of nutrient concentrations in both treatments solutions, DCAP leaves proved more efficient in the absorption of the above-mentioned elements. In the work of Delaide et al. (2016) with comparable experimental design concerning external inputs in the decoupled system, many macro- and micro- nutrients were substantially enhanced in leaf tissues of decoupled aquaponics compared to hydroponic plants (*e.g.*, P, K, Ca, Mg). Like these authors, we cannot directly explain these differential elements absorption on the basis of the measured parameters. Only speculation might be made on a possible contribution of microorganisms present in the fish water or the rhizosphere of DCAP plants as well as of dissolved organic molecules usually found in RAS water which might have promoted growth and/or nutrient acquisition (as per Monsees et al., 2019; Delaide et al., 2021). In the latter case, we may hypothesize that external fertilizer input might have potentiated these processes, since the same reaction was not recorded in CAP plants which also received the same fish water. Indeed, CAP plants contained significantly lower N, K, Fe, Zn and Cu in their leaves but higher P, Ca, and Mg. The sub-sufficient levels of important nutrients in leaf tissue of plants cultivated under coupled aquaponics has been repeatedly reported (Ru et al., 2017; Yang & Kim, 2019; Tsoumalakou et al., 2022). This system’s feature and the imposed productivity limitations drove to the development of decoupled systems as a measure to tackle the problem.

### Integrated picture of basil performance under the three treatments

According to the above discussion and if we try to interconnect the various functional responses encountered, the following integrated picture of basil performance arises. CAP plants have absorbed and assimilated substantially lower amounts of crucial macro- and micro-nutrients (*e.g.*, N, K, Zn and Fe) compared to the other treatments. This fact probably had important implications to chlorophyll biosynthesis, and virtually all aspects of photosynthetic function of CAP basils. Fe deficiency, for example, negatively affects photosynthetic electron transport due to high demands of the latter for Fe co-factors, given also that chloroplast is the main sink for Fe (Kroh & Pilon, 2020). Poor CAP leaf nutritional state may has also direct negative effects on photosynthesis, through another route, the well documented down-regulation by N-deficiency (Hermans et al., 2006; Del Pozo et al., 2007). The mechanism behind this effect relates to feedback regulation by sugar accumulation that also affects genes involved in photosynthesis. The sub-optimal nutrient status of CAP plants hampers growth, whereby weakening the sink strength which again induce a negative feedback on photosynthesis (Ruiz-Vera et al., 2017). The ultimate result of these impacts is the down-regulation of photosynthesis which was depicted not only in decreased instantaneous CO_2_ assimilation and PSII activity but also in the reduced concentrations of the photosynthetic pigments of CAP leaves. All the above may be considered an acclimation process of the photosynthetic apparatus to the prevailing nutrient conditions, in order to succeed coordination and balance between the best possible nutrient exploitation for growth and efficient light use efficiency under the given nutrient restrictions. Noteworthy to this direction is the enhanced root-to-shoot ratio of CAP plants which is also an acclimatory response to nutrient shortage. This biomass allocation pattern indicates that mineral elements are inadequate for optimal growth which triggers metabolic changes in the shoot resulting in increased carbohydrate transport to the root to facilitate nutrient foraging (Hermans et al., 2006).

## Conclusions

The functional responses of CAP plants were predominantly driven by sub-sufficient levels of crucial nutrients in leaf tissue. This result triggered a series of negative feedback loops on photosynthesis in terms of both instantaneous gas exchange and PSII photochemical efficiency. The resulting down-regulation of photosynthesis is connected also to decreased pigments content and whereby light use efficiency. All the above corresponds most probably to an acclimation process than to a functional impairment. The ultimate result, however, is the decreased growth of CAP plants.

DCAP plants outperformed all the others in terms of photosynthetic performance and growth, yet without consistent statistically significant differences with HP treatment. Their superiority against CAP plants may be ascribed to more sufficient nutrient concentrations in leaf tissues, which induced positive feedback to the efficiency of photosynthetic apparatus. In comparison to HP plants, DCAP basil reached the same or enhanced level of growth, photosynthetic efficiency and pigment content with lower mineral fertilizers input. In conclusion, decoupled aquaponic systems are favorable compared to the classical coupled systems and may also replace the conventional hydroponic systems in commercial facilities. The present study of basil functional responses to the three tested production systems provided insights on the underlying mechanisms of plant performance highlighting key-points for systems optimization. Nevertheless, since aquaponic systems are complex, dynamic and species-specific, more work on the mechanisms involved in crop and fish species function and interaction is needed to eliminate bottlenecks and optimize the aquaponic ecosystem productivity.

##  Supplemental Information

10.7717/peerj.15664/supp-1 Supplemental Information 1 Raw data

## Additional Information and Declarations

### Data Availability

The following information was supplied regarding data availability: The raw measurements are available in the Supplementary File.
